# RPTOR blockade suppresses brain metastases of NSCLC by interfering the ceramide metabolism via hijacking YY1 binding

**DOI:** 10.1186/s13046-023-02874-z

**Published:** 2024-01-02

**Authors:** Ying Lin, Yun Wu, Qiangzu Zhang, Xunwei Tu, Sufang Chen, Junfan Pan, Nengluan Xu, Ming Lin, Peiwei She, Gang Niu, Yusheng Chen, Hongru Li

**Affiliations:** 1 Department of Respiratory and Critical Care Medicine, Shengli Clinical Medical College, Fujian Medical University, Fujian Provincial Hospital, Fuzhou, 350001 Fujian China; 2 Department of General Practice Medicine, Fujian Provincial Hospital, Fuzhou, 350001 China; 3 The High Performance Computing Research Center, Institute of Computing Technology, Chinese Academy of Sciences, Beijing, 100095 China; 4 The Centre for Experimental Research in Clinical Medicine, Fujian Provincial Hospital, Fuzhou, 350001 Fujian China; 5 Fujian Provincial Key Laboratory of Medical Big Data Engineering, Fujian Provincial Hospital, Shengli Clinical College of Fujian Medical University, Fuzhou, 350001 Fujian China

**Keywords:** Non-small cell lung cancer, Brain metastasis, RPTOR, SPHK2, S1P

## Abstract

**Background:**

Ceramide metabolism is crucial in the progress of brain metastasis (BM). However, it remains unexplored whether targeting ceramide metabolism may arrest BM.

**Methods:**

RNA sequencing was applied to screen different genes in primary and metastatic foci and whole-exome sequencing (WES) to seek crucial abnormal pathway in BM + and BM-patients. Cellular arrays were applied to analyze the permeability of blood–brain barrier (BBB) and the activation or inhibition of pathway. Database and Co-Immunoprecipitation (Co-IP) assay were adopted to verify the protein–protein interaction. Xenograft and zebrafish model were further employed to verify the cellular results.

**Results:**

RNA sequencing and WES reported the involvement of RPTOR and ceramide metabolism in BM progress. RPTOR was significantly upregulated in BM foci and increased the permeability of BBB, while RPTOR deficiency attenuated the cell invasiveness and protected extracellular matrix. Exogenous RPTOR boosted the SPHK2/S1P/STAT3 cascades by binding YY1, in which YY1 bound to the regions of SPHK2 promoter (at -353 ~ -365 nt), further promoting the expression of SPHK2. The latter was rescued by YY1 RNAi. Xenograft and zebrafish model showed that RPTOR blockade suppressed BM of non-small cell lung cancer (NSCLC) and impaired the SPHK2/S1P/STAT3 pathway.

**Conclusion:**

*RPTOR* is a key driver gene in the brain metastasis of lung cancer, which signifies that RPTOR blockade may serve as a promising therapeutic candidate for clinical application.

**Supplementary Information:**

The online version contains supplementary material available at 10.1186/s13046-023-02874-z.

## Background

Brain metastasis (BM) is a major cause of poor prognosis, recurrences, and deaths in patients with non-small cell lung cancer (NSCLC), with an incidence rate of approximately 20–40% in the patient population [1–3]. Therefore, pinpointing key driver genetic events in the brain metastasis of lung cancer serves as the focus of recent research endeavors [4].

In tumor metastases, the mammalian target of rapamycin (mTOR) signaling pathway plays an important role and mTOR inhibitors have been clinically employed for the treatment of some metastatic tumors [5–8]. The mTOR signaling pathway consists of two functionally and structurally distinct multiprotein complexes, mTORC1 and mTORC2 [9, 10]. As an indispensable component of mTORC1, the regulatory-associated protein of mTOR (RPTOR) is a critical regulator of mTORC1. RPTOR is upregulated in a variety of cancers and promotes tumor metastasis in various ways. However, it remains blurred with regards to the association of RPTOR with the brain metastasis of lung cancer.

*RPTOR* is located on human autosomal 17q25.3 and encodes RPTOR protein with 1335 amino acids and its coding region contains 34 exons. Several studies suggest that an abnormally-elevated expression of RPTOR may be closely associated with tumor metastasis [11–13]. A previous study documents that the brain metastasis of lung cancer is associated with the abnormality of the PI3K-PTEN-AKT-MTOR signaling pathway induced by the genetic polymorphism of oligonucleotide SNP [14]. A more recent study demonstrates that an increased risk of brain metastasis is closely linked to the mutation of mLST8 [15]. As mLST8 and RPTOR are important components of mTORC1, these findings strongly suggest that RPTOR might be implicated in the brain metastasis of lung cancer.

RPTOR positively regulates the activity of mTORC1, which is related to lipid metabolism, while ceramide is one of the important metabolites of sphingolipids [16–19]. Recent findings suggest that the mTOR signaling pathway may be related to the activation of sphingosine kinases (SPHK), SPHK1 and SPHK2, which promote the metabolization of ceramide into sphingosine-1-phosphate (S1P) [20]. Studies show that the up-regulation of SPHK2 is associated with growth and metastasis in various cancers [21] and that the overexpression of SPHK2 can inhibit the cellular apoptosis of NSCLC, suggesting that the activity of SPHK2 is related to the development and prognosis of lung cancer [22]. S1P, in turn, mediates tumor growth and metastasis via S1P receptor 1 (S1PR1)-dependent or S1PR1-independent signaling pathways [23]. The upregulation of S1PR1 can activate STAT3, which binds to the promoter of S1PR1 to regulate its transcription, forming a positive feedback loop and accelerating tumor growth and metastasis [24]. Evidence indicates that the activation of the S1PR1-STAT3 signaling pathway mediates the onset of intracranial tumor metastasis [24, 25]. Therefore, we hypothesized that RPTOR may promote the brain metastasis of lung cancer through the SPHK2/S1P/STAT3 axis.

In this study, we conducted in vitro and in vivo experiments with samples of NSCLC to investigate the effects of RPTOR expression on its brain metastasis. We found that RPTOR promoted the cerebral invasion of the NSCLC lung cancer cells by the SPHK2/S1P/STAT3 axis. The findings provide novel insights into the role of *RPTOR* in tumor metastases.

## Materials and methods

### Patient samples

Paraffin-embedded paired samples of lung primary cancer and BM tissues from lung adenocarcinoma (LUAD) patients with BM who were admitted to Fujian Provincial Hospital from January 2011 to December 2019 were retrospectively collected. Tissue samples were classified according to the World Health Organization (WHO) Histological Classification of Lung and Pleural Tumors, fourth edition, and patients diagnosed based on the 2018 International Association for the Study of Lung Cancer (IASLC) Pathology IALIS Diagnostic criteria. HE-stained slides of pathological tissues before and after metastases were reviewed in a double-blind manner by two experienced pathologists who had access to the complete clinicopathological data and survival-related information of these patients. The study protocol was approved by the Ethics Committee of Fujian Provincial Hospital.

### RNA sequencing and data analysis of paired lung primary cancer and BM tissues

Three paraffin-embedded paired lung primary cancer and BM tissues were selected from above samples. RNA isolation, cDNA library construction, and RNA sequencing were performed by GENEWIZ Biotechnology Co. (Suzhou, China), Ltd. An mRNA library was constructed using the Illumina Truseq mRNA Strand Sample Prep Kit (San Diego, California, USA), according to the manufacturer's instructions. The cDNA libraries were sequenced on an Illumina Hiseq 2500 sequencer (San Diego, California, USA).

The imaging data of sequences measured with the high-throughput sequencer were converted into sequence-based data (reads) by CAsSAVA base recognition. Files were stored in FASTQ format, and gene expression was quantified as fragments per kilobase million (FPKM). mRNA annotations in the human genome were retrieved from the GENCODE (V25) database. Gene expression in two groups was compared by t-tests, with > 1.5 fold changes and adjusted *P* values < 0.05 considered statistically significant. Upregulated genes were identified by volcano plots, with log two-fold change as the abscissa and -log adjusted P-value as the ordinate.

### Framework of damage assessment of genomic mutations (DAGM) model

To explore the differences in signaling pathways between LUAD with and without BM, 69 LUAD patients with BM (BM +) and 82 LUAD patients without BM (BM-) were recruited. Peripheral blood or tissue samples were obtained from these patients for whole-exome sequencing (WES). Based on variant allele frequency (VAF), these samples were categorized as Classes 1 (leaf clone), 2 (branch clone), and 3 (trunk clone). A DAGM model was constructed to assess the role of somatic variants in signaling pathways. The mutated somatic variants from every clone or clonal composition were mapped to the 60 signaling pathways to obtain composite scores of signaling pathways in different forms. Ceramide signaling pathways with different clonal distributions were also compared between BM + and BM- samples by Activity Profiles of Signalling Pathways (APSPs) [26].

### HE and IHC staining

Primary lung cancer and BM tissues were histopathologically stained with hematoxylin–eosin (HE) and immunohistochemically stained with antibodies to RPTOR, Ki67, CD34, and MMP9 that were provided by Abcam Plc Co., Ltd. (Cambridge, UK), according to the manufacturer’s instructions. All tissue samples were scored as 0 (no staining), 1 (weak, incomplete membrane staining < 10%), 2 (moderate, complete membrane staining from 11 to 50%), 3 (strong, complete homogenous membrane staining from 51 to 80%) or 4 (complete homogenous membrane staining more than 80%). Tissues with RPTOR scores ≥ 6 and ≤ 3 were defined as the high and low RPTOR-expressing groups, respectively [27].

### Culture and treatment of cell lines

Human LUAD cell lines (A549, PC9 and HCC827), a human NSCLC cell line (H1299), a lung squamous cell carcinoma cell line (H226) and a human lung epithelial cell line (BEAS-2B) were purchased from the Cell Resource Center of the Institute of Basic Medical Sciences, Chinese Academy of Medical Sciences. A human brain microvascular endothelial cell line (hCMEC/D3) was purchased from Meisen Cell Technology Co., Ltd. (Zhejiang, China), and a mouse astrocyte cell line (CP-M157) was obtained from Procell Co., Ltd. (Wuhan, China). RPTOR shRNA, a RPTOR overexpressing lentivirus, their corresponding controls, and a firefly luciferase reporter gene plasmid with SPHK2 promoters and genes encoding for puromycin resistance were obtained from Hanheng Biological Co., Ltd. (Guangzhou, China). After lentiviral infection, the cells were cultured in a CO_2_ incubator, using puromycin to screen stable cell lines for subsequent in vitro and in vivo experiments.

### Quantitative reverse transcription-polymerase chain reaction (qRT-PCR)

RNA was isolated and extracted from tissue samples and cells using TRIzol reagents. PCR was performed with the PrimeScript RT Kit. GAPDH was used as an internal control. RNA isolation and extraction were conducted from tissue samples and cellular specimens utilizing TRIzol reagents. Subsequently, reverse transcription was employed to synthesize complementary DNA (cDNA). Polymerase Chain Reaction (PCR) was executed using the PrimeScript RT Kit. Glyceraldehyde-3-phosphate dehydrogenase (GAPDH) served as an internal control. The primer sequences were as follows: RPTOR: Forward GACACGGATGTTCGACAAG, Reverse ATCTGAGAAGCAACGCTCC; GAPDH: Forward CAACGTGTCAGTGGTGGACCTG, Reverse GTGTCGCTGTTGAAGTCAGAGGAG; YY1: Forward CCTGGCATTGACCTCTCAGATCCCA, Reverse GGGCAAGCTATTGTTTTGGAGCA; SPHK2: Forward TTCTATTGGTCAATCCCTTTGG, Reverse AGCCCGTTCAGCACCTCA.

### Western blotting

Western blotting experiments were performed as previously described. The primary antibodies used in the western blotting were as follows: RPTOR (1:1000, ab40768; Abcam), YY1 (1:1000, #46395; Cell Signaling Technology [CST]), Ki67 (1:1000, ab16667; Abcam), CD34 (1:1000, ab81289; Abcam), MMP9 (1:1000, ab228402; Abcam), MMP2 (1:1000, ab92536; Abcam), SPHK2 (1:1000, ab264042; Abcam), S1P1 (1:1000, ab233386; Abcam), Stat3 (1:1000, #9139; CST),Phospho-Stat3 (Tyr705) (1:1000, #9145; CST), Phospho-Stat3 (Ser727) (1:1000, #94994; CST), E-cadherin (1:1000, #14472; CST), N-cadherin (1:1000, #13116; CST), Vimentin (1:1000, #5741; CST), Snail (1:1000, #3879; CST), Slug (1:1000, #9585; CST), GAPDH (1:5000, 10494–1-AP; Proteintech), ZO-l (1:1000, #13663; Cell Signaling Technology), Occludin (1:1000, #91131; CST), Claudin 5 (1:1000, ab131259; Abcam). The secondary antibodies used in the western blotting were as follows: HRP- Goat Anti-Rabbit JgG (Immumoway); HRP- Goat Anti-mouse IgG (Immumoway).

### Co-Immunoprecipitation (Co-IP) assays

Cells were lysed in RIPA buffer, and the cell lysates were clarified by centrifugation at 14,000 × g for 10 min. The supernatants were collected, and a 2 µg aliquot of each primary antibody was added to 1 mg of clarified total cell lysate. The mixtures were incubated overnight at 4 °C, followed by the addition of protein agarose beads and incubation for 2 h. The beads were washed three times with icecold RIPA buffer, followed by the addition of 1 × SDS loading buffer. The samples were microcentrifuged for 30 s, and the supernatants heated to 96 °C for 10 min and centrifuged for 1 min at 14,000 × g. In addition, the temperature of centrifugation was 4 °C. The protein samples were loaded onto SDS-PAGE gels and analyzed by western blotting [28].

### Construction of a blood–brain barrier (BBB)

An in vitro BBB model was constructed as previously described [29, 30]. Briefly, hCMEC/D3 cells and astrocytes were co-cultured on opposite sides of a 24-well transwell polycarbonate insert, with 10^5^ PC9 cells expressing EGFP in 1 mL hCMEC/D3 medium placed in the upper chamber, and mouse CP-M157 astrocytes in astrocyte medium placed in the lower chamber; due to the COVID-19 policy in China at that time, human astrocyte cell lines could not be obtained. After 24 h, the cells in the lower chamber were washed with PBS and fixed with 4% paraformaldehyde at room temperature for 20 min. The cells were photographed under a fluorescence microscope, and the average number of EGFP-positive migrated cells counted in five random fields. The absorbance of the culture medium at 450 nm was measured, and permeability was calculated using the formula: PHRP% = (CHRP lower chamber × VHRP lower chamber)/(CHRP upper chamber × VHRP upper chamber) × 100%.

#### Dual-luciferase reporter assays

The binding of transcription factors (reporter gene) to the promoter of downstream target genes were analyzed by dual-luciferase reporter assays. Briefly, a full-length luciferase reporter gene of the SPHK2 promoter was constructed by Fuzhou Zaiji Biotechnology Co., Ltd. (Fuzhou, China), and dual-luciferase reporter assays performed using specific kits. All experiments were performed in triplicate.

#### Chromatin Immunoprecipitation (ChIP)

Fixed 1 × 10^7^ cells with formaldehyde and glycine. Lysed cells using membrane extraction buffer, fragmented DNA with MNase and sonication, confirmed by agarose electrophoresis. DNA fragments underwent overnight incubation with antibodies. Purified antibody-DNA complexes with Protein A/G magnetic beads and elution buffers. Analyzed enriched DNA fragments by qRT-PCR. Antibody used: YY1 (#63227, CST) [31].

#### Construction of BM model in mice and zebrafish

The BM model of lung cancer was constructed as previously described [32]. Briefly, after the tumorigenesis in mice, the animals were divided into the following groups on the basis of the lentivirus vectors transfected into the PC9 cells: the lentivirus carrying the *RPTOR*-knockdown group (shRPTOR2#) and corresponding negative control group (shNC), and the lentivirus carrying the *RPTOR*-overexpression group (OE) and corresponding control group (VE). Under a stereo microscope, 50 μL of Luc-PC9 cells at a concentration of 3 × 10^5^ cells in 0.1 mL PBS were slowly injected into the carotid artery of nude mice. After about 8 weeks, or when the mice displayed clinical BM symptoms, such as slow movement, weight loss and hunchback, bioluminescence imaging was performed. Briefly, fluorescein, at a concentration of 150 mg/kg, was intraperitoneally injected into each mouse. About 15–20 min later, the IVIS Lumina X5 imaging system (PerkinElmer imaging, USA) was employed to quantify regions of interest (ROI) of the animals. The mice were subsequently euthanized and their brains removed and cut into thin slices (2 to 3 mm). The slices were histopathologically examined by staining with hematoxylin–eosin (HE) and were evaluated immunohistochemically by staining with specific antibodies.

To observe whether abnormal RPTOR expression promotes BM in the host, the zebrafish BM model was established by overexpressing or knocking down *RPTOR* in zebrafish eggs and subsequently injecting lung cancer cells into the embryos, as previously described [33, 34]. Briefly, zebrafish eggs at the unicellular stage of the Tg (coro1a:EGFP) line were recruited. Different dispositions were applied for zygotic injection and the eggs were divided into the following five groups: the blank control group (blank), *RPTOR*-overexpressed group (RPTOR OE) and corresponding control group injected with H_2_O (H_2_O) and *RPTOR*-knockdowned group (RPTOR KD) and corresponding control group injected with CasRx (CasRx). Next, H1299 and PC9 cells were respectively microinjected at 48 hpf into the perivitelline space of zebrafish embryos, each embryo carrying 150–200 cancer cells. The embryos were cultured in accordance with standard procedures at 28.5 ºC. They were chosen and photographed at days 1 and 3 after injection by fluorescent stereomicroscopy (SMZ800N, Nikon, Japan) and the number of tumor cells calculated.

#### Statistical analysis

All statistical analyses and mapping were performed using SPSS 20.0, Image J, and GraphPad Prism 8, R (v3.6.3) software. The statistical methods used to compare two groups in our article were t-test and Kruskal-Wallis test, and t-test was used when the number of group sample was > 50. Survival was analyzed using the Kaplan-Meier method and compared by log-rank tests. *P* values < 0.05 were defined as statistically significant.

In the zebrafish experiments, cartograms were created by GraphPad, and fluorescent images were obtained by ImageJ software. Results were reported as the mean ± the standard error of the mean (SEM) and compared by t-tests.

## Results

### RPTOR is highly expressed in the brain metastatic human LUAD and is associated with poor prognosis

**Fig. 1:**
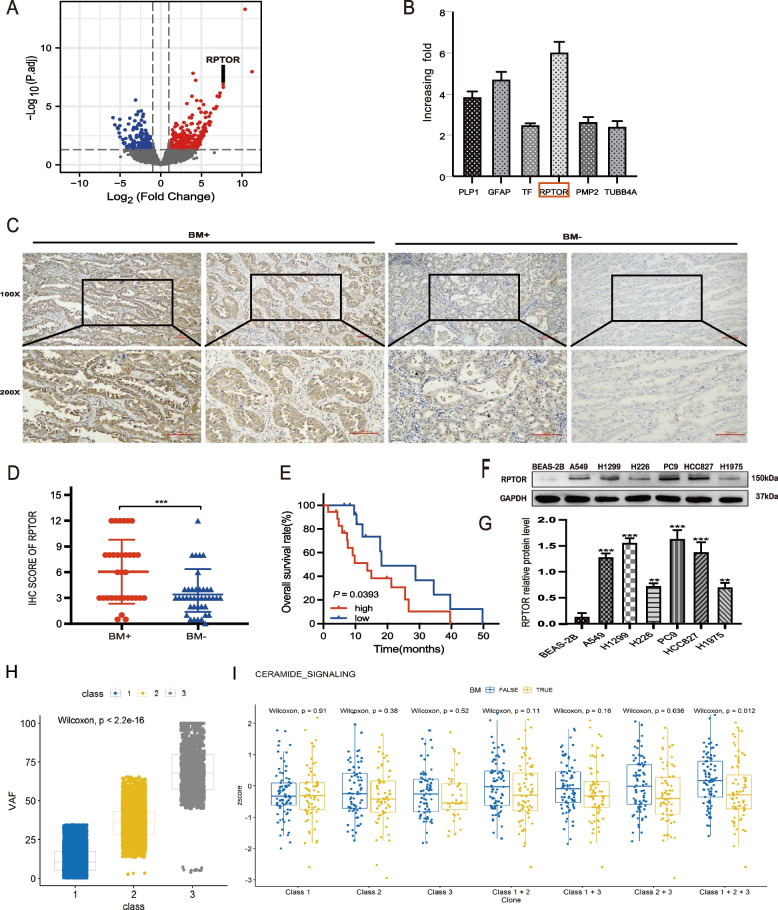
Association of high RPTOR expression or the ceramide pathway with NSCLC-BM. **A**, **B** RNA sequencing showed that *RPTOR* was more highly expressed in BM than other genes like *PLP1*, *GFAP*, *TF*, *PMP2* and *TUBB4A*, which was validated by qRT-PCR. **C**, **D** IHC analysis showed that the BM + group reported a higher RPTOR expression and score than the BM- group (100x, 200 × magnification, scale bar = 100 μm). **E** Kaplan–Meier analysis showed that the median survival was significantly lower in the high RPTOR (18 samples with IHC scores ≥ 6) than in the low RPTOR subgroup (15 samples with IHCs ≤ 3) in the BM + patients. **F**, **G** H1299 and PC9 cell lines were chosen to be our experiment cell lines due to their significantly higher RPTOR expression than those in other LUAD cell lines. **H**, **I** The whole-exome sequencing (WES) was used to screen different genes from the BM + and BM- groups. A DAGM model was constructed to assess the role of somatic variants in the signaling pathways. The ceramide signaling pathway was found to be involved in the BM progress

The LUAD specimens were divided into two groups. The BM + group consisted of 33 tissue samples, 12 primary lung tumor and 21 BM samples, including three sets of paired primary tumor and BM tissue samples from the same LUAD patients; whereas the BM- group consisted of 40 primary lung tumor samples. RNA sequencing analysis of the three pairs of tissue samples identified several relevant candidate genes related to BM. The top ranked up-regulated genes in BM included *PLP1*, *GFAP*, *TF*, *RPTOR*, *PMP2* and *TUBB4A* (Fig. 1A). The results showed that RPTOR mRNA was significantly up-regulated in the metastatic foci when compared with primary pulmonary foci (Fig. 1B), suggesting that RPTOR expression might be linked to NSCLC-BM.

The analysis of The Cancer Genome Atlas (TCGA) database showed that the high RPTOR expression was associated with a poor prognosis in patients with NSCLC (Supplementary Fig. S1A, B, C). The present study found that RPTOR was highly expressed in the cytoplasm of primary tumors and BM tissues, with RPTOR IHC scores higher in LUAD patients with than those without BM (Fig. 1C, D). Kaplan–Meier survival analysis showed that the median survival was significantly shorter in patients expressing a high level of RPTOR than in those with a low level (19.8 vs. 34.5 months, *P* = 0.039) (Fig. 1E), further suggesting that a high RPTOR expression is associated with a poor prognosis in the LUAD-BM patients.

Western blotting showed that the RPTOR expression was higher in the NSCLC cell lines (H1299 and PC9) than in other LUAD cell lines, such as A549, HCC827, and H1975 cells, or than in the squamous cell carcinoma line H226, and was the lowest in the normal lung epithelial cell line BEAS-2B (Fig. 1F, G). Therefore, further experiments in this study were performed using H1299 and PC9 cells [35, 36].

### The down-regulation of ceramide pathways is associated with LUAD-BM

The variant allele frequencies (VAFs) of the three clonal populations differed significantly from one another. In each patient, the tumor cell population primarily comprised trunk and branch clones (Classes 2 and 3) (Fig. 1H). The activation of Class 2 + 3 and Class 1 + 2 + 3 ceramide pathways was markedly down-regulated in the BM + group, suggesting that the reduced activation of ceramide pathways was closely associated with the BM of lung cancer (Fig. 1I). Taken together, these findings indicate that the down-regulation of the ceramide pathway might induce the BM of the lung cancer.

### RPTOR increases the permeability of BM of human LUAD in vitro

**Fig. 2:**
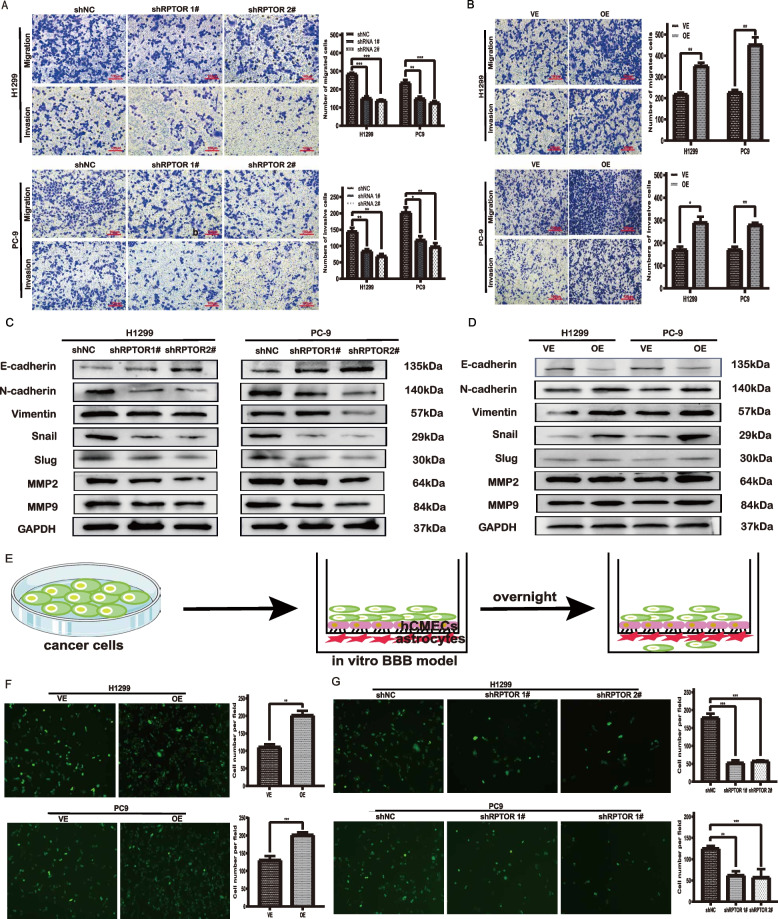
The RPTOR-promoted traverse of lung cancer cells to the BBB. **A**, **B** Transwell assays showed that *RPTOR* promoted the migration and invasion of lung cancer cell lines. **C**, **D** Western blotting reported that *RPTOR* increased EMT-related proteins and extracellular matrix. **E**, **F**, **G** Fluorescence microscope transwell assays showed that *RPTOR* promoted the traverse of lung cancer cells to the BBB

After RPTOR interference or overexpression, the in vitro assays, including Cell Counting Kit-8 (CCK8), clone formation assay, and wound healing, reported that *RPTOR* positively impacted the migration, invasion and proliferation of lung cancer cell lines (Supplementary Fig. S2A-L). Transwell assays also showed that *RPTOR* expression was positively correlated with the migration and invasion of lung cancer cell lines (Fig. 2A, B). Western blotting showed that RPTOR expression also positively impacted the expression of epithelial-mesenchymal transition (EMT)-related proteins, including E-cadherin, N-cadherin, vimentin, snail, slug, and matrix metalloprotein 9 (MMP9) (Fig. 2C, D).

The effect of RPTOR expression on the ability of lung cancer cells to penetrate the BBB was assessed by coculturing hCMEC/D3 cells and astrocytes on opposite sides of a 24-well transwell polycarbonate insert (Fig. 2E). RPTOR overexpression markedly promoted the cellular penetration of the BBB (Fig. 2F), which was significantly reversed by RPTOR knockdown (shRPTOR1# and shRPTOR2#) (Fig. 2G). These findings indicate that RPTOR enhances the ability of lung cancer cells to traverse the BBB.

### RPTOR promotes the BM of LUAD via the SPHK2/S1P/STAT3 axis

**Fig. 3:**
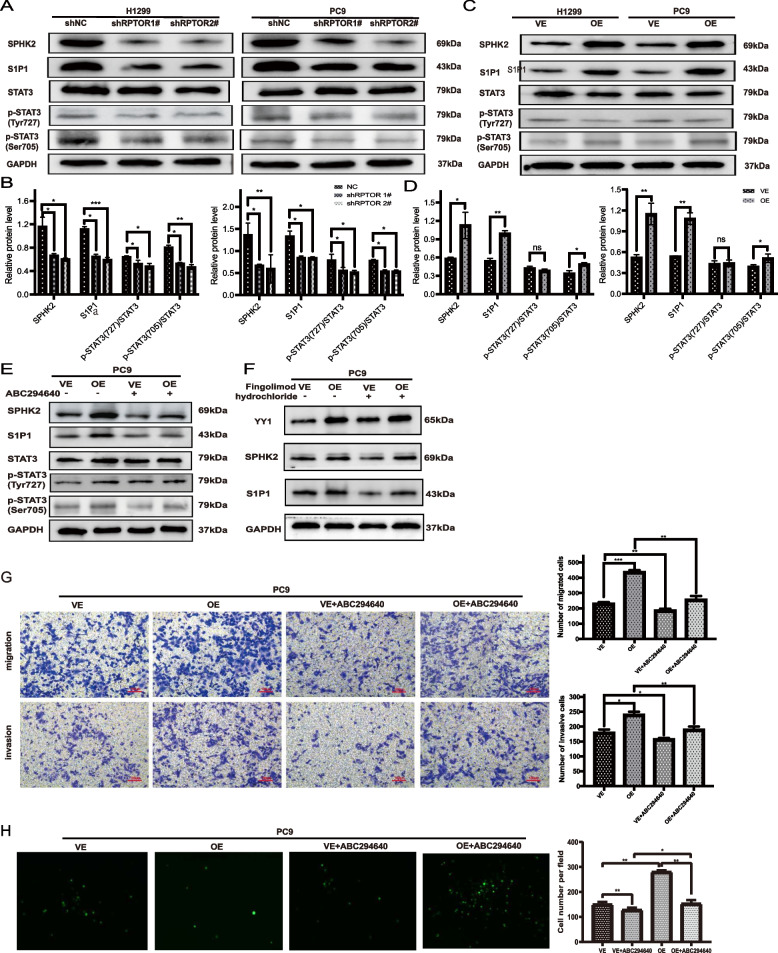
The RPTOR-promoted BM of NSCLC through the SPHK2/S1P/STAT3 signaling pathway. **A**-**D** Western blotting showed that the exogenous RRTOR boosted the expression of proteins of the ceramide metabolism pathway, including SPHK2, S1PR1 and p-STAT3 (ser705). **E**–**H** The SPHK2 inhibitor, ABC294640, reversed the activation of the SPHK2/S1P/STAT3 signaling pathway and the RPTOR overexpression-increased cell invasiveness and permeability of BBB, while the S1P inhibitor, fingolimod hydrochloride, reduced the expression of S1P1 protein, without affecting the expression of YY1 and SPHK2

The gene set enrichment analysis (GSEA) and Kyoto Encyclopedia of Genes and Genome (KEGG) pathway analysis were conducted to examine the RNA sequencing results of the LUAD patients with and without BM. The results showed that *RPTOR* was positively associated with the sphingomyelin pathway (*P* = 0.006) (Supplementary Fig. S3A), especially with SPHK2 (Supplementary Fig. S3B). Western blotting showed that proteins on the signaling pathway, such as SPHK2, S1PR1 and p-STAT3 (ser705), were markedly downregulated by RPTOR knockdown (Fig. 3A, B), but significantly enhanced by RPTOR overexpression (Fig. 3C, D). The addition of the SPHK2 inhibitor, ABC294640, to the RPTOR-overexpressed PC9 cells significantly inhibited the *RPTOR*-induced activation of the SPHK2/S1P/STAT3 signaling pathway (Fig. 3E) and restrained the RPTOR overexpression-enhanced proliferation, migration and invasion of lung cancer cell lines (Supplementary Fig. S4A, B). However, the S1P inhibitor, fingolimod hydrochloride, did not alter the expressions of YY1 and SPHK2, which were upregulated by RPTOR overexpression (Fig. 3F). The SPHK2 inhibitor, ABC294640, also reversed the RPTOR overexpression-increased migration and invasion of lung cancer cell lines and the permeability of BBB models (Fig. 3G, H). These findings evidence that RPTOR can promote the metastasis of NSCLC through the SPHK2/S1P/STAT3 signaling pathway.

### RPTOR upregulates SPHK2 by binding to transcription factor YY1

**Fig. 4:**
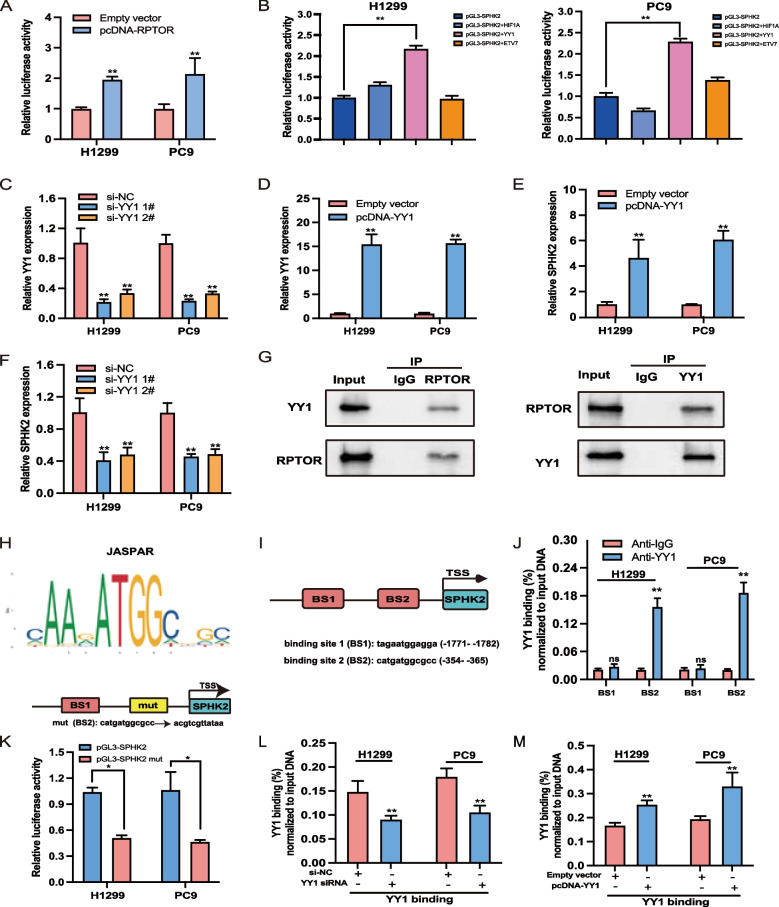
The RPTOR-regulated SPHK2/S1P pathway via binding to transcription factor YY1. **A** Dual-luciferase reporter assays indicated that *RPTOR* enhanced SPHK2 expression in H1299 and PC9 cell lines. **B** Transcription factor YY1 was screened to significantly enhance the transcriptional activity of SPHK2 when compared with other candidate transcription factors like HIF1A and ETV7, when co-transfected with pGL3-SPHK2. **C**-**F** qRT-PCR assays revealed a positive correlation between SPHK2 and YY1 expression, with both YY1 and SPHK2 expression down-regulated after treatmenting PC9 and H1299 cells with pcDNA-YY1 or YY1 siRNA. **G** Co-IP assays showed mutual interactions between RPTOR and YY1 in lung cancer cell lines. **H**–**K** The JASPAR database was used to predict YY1-binding sites in the SPHK2 promoter sequences. BS1 and BS2 were predicted to be YY1-binding sites, with BS2 at -353 ~ -365 nt of SPHK2 reporting a greater DNA enrichment by ChIP assays. The enrichment of SPHK2 DNA was reduced by mutation of BS2, confirming that BS2 is the site in the SPHK2 promoter bound by YY1. **L**-**M** Effect of YY1 expression on SPHK2 promoter sequences. YY1 enriched fewer SPHK2 promoter sequences after si-YY1 1# transfection but more sequences after pcDNA-YY1 transfection

Analyses of the Search Tool for the Retrieval of Interacting Genes (STRING) and Gene Expression Profiling Interactive Analysis (GEPIA) databases reported a close association of *RPTOR* with SPHK2 and the transcription factor, YY1. Cytoscape software analysis of the BioGrid database showed that three transcription factors, HIF1A, ETV7, and YY1, were most tightly correlated with the RPTOR-protein interaction network. The JASPAR database showed that these transcription factors were bound to promoter sequences 2 kb upstream of the SPHK2 pathway. Dual-luciferase reporter assays found that pcDNA-RPTOR promoted the SPHK2 expression (Fig. 4A). Of these three overexpressed transcription factors (HIF1A, YY1, and ETV7), YY1 significantly contributed to the SPHK2 expression (Fig. 4B). The treatment of PC9 and H1299 cells with pcDNA-YY1 or YY1 siRNA revealed a positive correlation between SPHK2 expression and YY1, as shown by qRT-PCR assays (Fig. 4C-F). Finally, Co-IP assays showed mutual interactions between RPTOR and YY1 in these lung cancer cell lines (Fig. 4G).

The JASPAR database predicted that YY1 was bound to the BS1 and BS2 sequences of the SPHK2 promoter (Fig. 4H, I). ChiP assays showed that a higher enrichment of BS2 DNA, located at -353 ~ -365 nt in the SPHK2 promoter (Fig. 4J). Dual-luciferase reporter assays found that mutated BS2 reduced the SPHK2 expression, confirming that BS2 of the SPHK2 promoter was the YY1-binding site (Fig. 4K). Transfection of si-YY1 1# reduced the number of SPHK2 promoter sequences (Fig. 4L), which was reversed by transfection of pcDNA-YY1 (Fig. 4M). These findings suggest that YY1 can positively modulate SPHK2 expression by binding to the SPHK2 promoter region.

### RPTOR blockade suppresses the BM of NSCLC and attenuates the SPHK2/S1P/STAT3 pathway in xenograft and zebrafish model

**Fig. 5:**
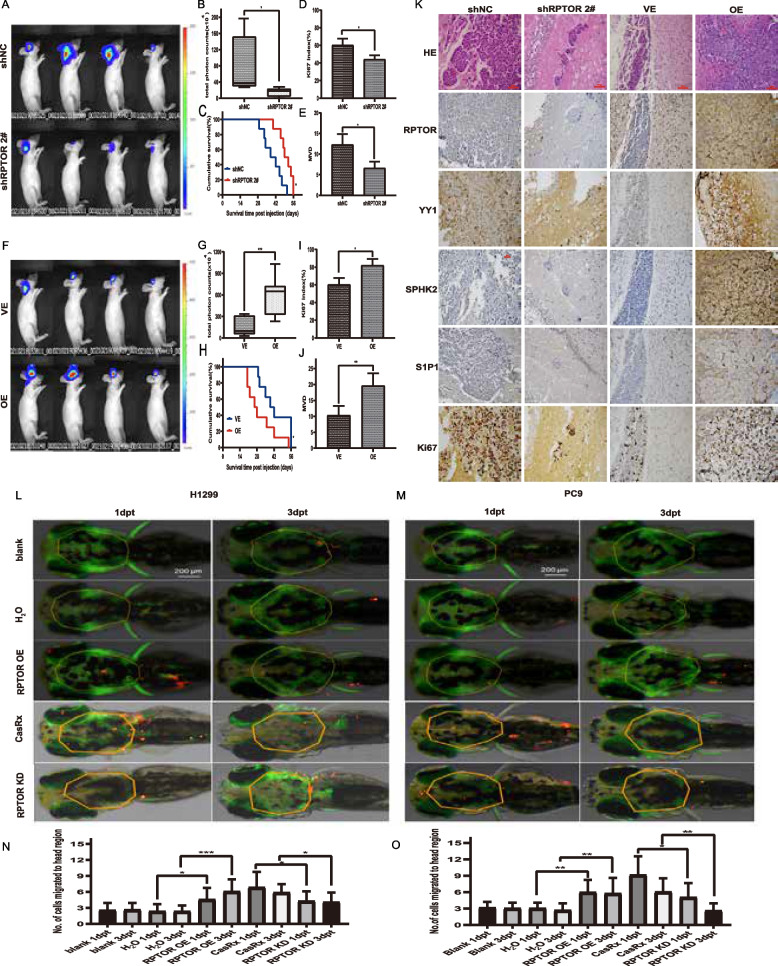
The suppressed BM of NSCLC and attenuated SPHK2/S1P/STAT3 pathway in xenograft and zebrafish model by RPTOR blockade. **A**-**J** PC-9 cells were injected intravenously to construct the BM mouse model. Fluorescence microscopy showed that RPTOR blockade significantly suppressed the BM formation, the fluorescence of tumor cells in the ROI of BM, the expression of the proliferation marker Ki67 and MVD in the peritumoral area, prolonging overall survival (200 × magnification, scale bar = 50 μm). RPTOR overexpression produced the opposite effects. **K** Effects of RPTOR expression on the expression of proteins in BM tissue samples in nude mice. RPTOR knockdown reduced the expressions of RPTOR, YY1, SPHK2, and S1P1, with RPTOR overexpression yielding the opposite effects (200 × magnification, scale bar = 50 μm). **L**-**O** H1299 and PC9 cells bearing vectors for RPTOR knockdown or overexpression were injected into the perivitelline space of zebrafish embryos. The number of cancer cells at days 1 and 3 after the injection was significantly higher in the RPTOR-overexpressed group than in their respective control groups, whereas the number of cancer cells at days 1 and 3 after injection was significantly lower in RPTOR-knockdown cells than in control cells

To construct in vivo models of lung cancer BM, nude mice were intravenously injected with human lung cancer cells. Compared with the shNC mice, the shRPTOR 2# mice reported a significant decrease in BM formation (Fig. 5A) and tumor cells in the BM ROIs, and a markedly-prolonged overall survival (OS) (Fig. 5B, C). These effects were reversed by RPTOR overexpression (Fig. 5F, G, H).

RPTOR knockdown reduced the expression of the proliferation marker Ki67 in tumor tissues and CD34-positive microvessel density (MVD) in the peritumoral area (Fig. 5D, E). These effects were reversed by RPTOR overexpression (Fig. 5I, J). HE staining and IHC showed that, compared with control mice (shNC), RPTOR knockdown (shRPTOR 2#) also significantly reduced the expressions of RPTOR and proteins of the SPHK2/S1P/STAT3 pathway in the brain tissues of nude mice, with RPTOR overexpression yielding the opposite effects (Fig. 5K).

To check whether abnormal RPTOR expression features a BM disposition in host, RPTOR was successfully overexpressed or knocked down in the zebrafish eggs (Supplementary Fig. S5A, B). Similar results were replicated in the established zebrafish BM model. Compared with the control, the number of cancer cells passing through the BBB at days 1 and 3 was significantly higher in cells overexpressing RPTOR (OE) and lower in cells with RPTOR knockdown (Fig. 5L-O).

## Discussion

It has been estimated that about 50% of patients with advanced NSCLC will develop BM, a clinical situation closely associated with a poor prognosis. However, the mechanisms underlying the BM development in these patients remain unclear.

In order to explore the primary cause of BM, we applied RNA sequencing to screen different genes in primary and metastatic foci and WES to pinpoint the crucial abnormal pathways in BM + and BM- patients. The screening results indicated *RPTOR* as a novel driver gene and ceramide pathway as a crucial abnormal signaling pathway involved in BM progression.

As a vital component of the mechanistic target of rapamycin complex (mTORC), RPTOR participates in several cellular processes, including protein and ribosome synthesis, cell autophagy [37, 38], and aggravates the advance and metastasis of tumors [39–44]. Although mTOR inhibitors are effective in inhibiting the growth of some tumors, previous clinical trials of the drugs have not yet yielded favorable results for NSCLC [45, 46].

**Fig. 6:**
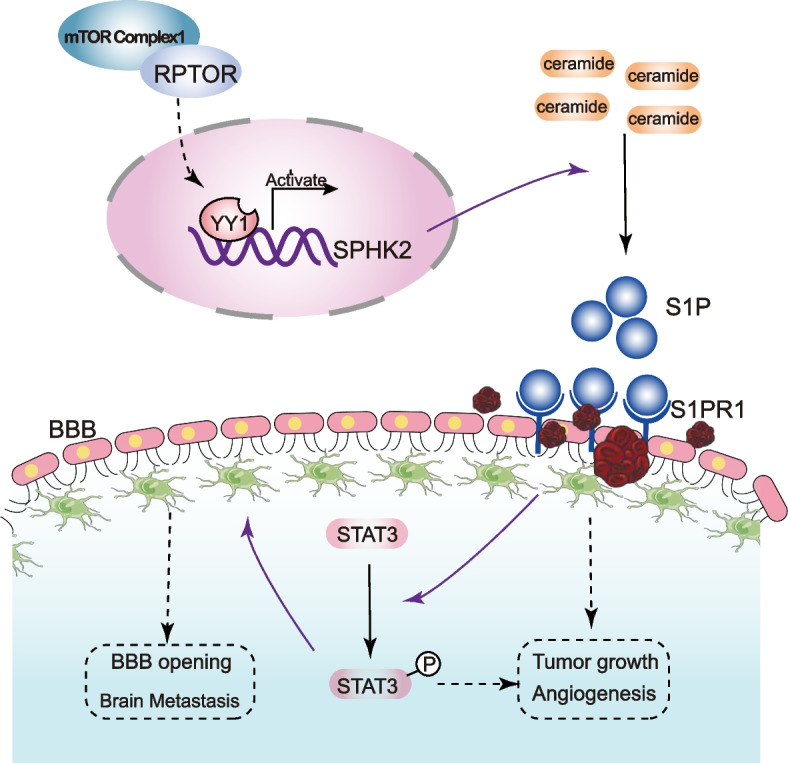
The mechanism underlying the RPTOR-promoted NSCLC-BM via the SPHK2/S1P/STAT3 signaling pathway. *RPTOR* regulated the transcriptional activation of the ceramidase SPHK2 by binding to the transcription factor YY1. SPHK2 catalyzed the degradation of ceramide to S1P. The latter, in turn, activated the STAT3 signaling pathway by binding to S1PR1 (solid lines), altering the microenvironment and enhancing the permeability of the BBB (dotted lines), resulting in the BM of NSCLC

To date, the mTOR signaling pathway may be involved in the BM progression. Studies have documented that mTORC1 can be positively regulated by RPTOR and is associated with the metabolism of lipids, such as ceramide [16–19]. As a critical sphingolipid metabolite, ceramide has recently been reported to be closely related to BM [4] and can be degraded to S1P by SPHK. Other studies have reported that SPHK2/S1P can impact the proliferation and metastasis of various cancers [35, 36] and that the combination of S1P and S1PR1 can activate STAT3, with the latter, in return, positively regulating the transcription of S1PR1, which persistently activates STAT3, setting in motion a positive feedback cycle and altering the microenvironment and permeability of the BBB, so as to enhance tumor growth and BM metastasis [47–49] On the basis of these findings, we hypothesize that *RPTOR* might boost the SPHK2/S1P/STAT3 cascades and promote the BM (Fig. 6).

The current study revealed that RPTOR participated in BM progress. The results showed that RPTOR significantly accumulated in brain metastatic foci and was related to the prognosis in BM of LUAD and that RPTOR overexpression increased the permeability of the BBB, while RPTOR deficiency impaired the cell invasiveness and protected extracellular matrix. In the mouse and zebrafish models, the respective RPTOR interference applied to the tumor cells and the host revealed that RPTOR blockage significantly suppressed the BM progress. These findings suggest that *RPTOR* may be a key driver gene in LUAD-associated BM.

Additionally, the mechanism underlying the RPTOR-boosted SPHK2/S1P/STAT3 cascades was explored. JASPAR database was utilized to predict some crucial transcription factors. The screening reported YY1 as the key agent in the RPTOR binding and function. According to ChiP assays, YY1 bound to the regions (at -353 ~ -365 nt) of the SPHK2 promoter and positively regulated the expression of SPHK2, which was rescued by YY1 RNAi. These finding indicate that *RPTOR* may regulate the SPHK2 signaling by binding to YY1. Collectively, these results evidence that RPTOR blockade can suppress the BM of NSCLC by attenuating the SPHK2/S1P/STAT3 pathway via hijacking the YY1 binding.

Given that the incidence of BM is higher in patients with LUAD than in those with squamous cell carcinoma, the present study analyzed the mechanism of the BM formation in LUAD. Two NSCLC cell lines, H1299 (EGFR wild type, p53-deficient) and PC9 (EGFR exon 19 deletion), were chosen in our study. Both of them showed a high RPTOR expression and were prone to BM. In our study, the identified driver genes and signaling pathways of BM were not significantly associated with EGFR mutations (data not shown). Therefore, *RPTOR* may be a novel critical driver gene of BM, independent of *EGFR* and other driver genes.

Until recently, effective treatments for BM remain limited. Although some patients with specific gene mutations may benefit from certain targeted therapies, traditional chemoradiotherapies still fail to provide satisfactory survival outcomes. Research should be encouraged to seek novel driver genes for BM development. Well-documented as it may be, the involvement of RPTOR in the BM of lung cancer has been barely reported. In our study, we first screened this gene as a novel BM-related driver gene and clarified the underlying mechanism, that is, RPTOR might promote BM via interfering the ceramide metabolism, which is also firstly proposed as a crucial signaling pathway of BM. Although available literature reports no positive results in phase-1 clinical trials of mTOR inhibitors for NSCLC, no differentiation has been made between patients with high and low RPTOR expression. In addition, an article published by Nature in 2022 has reported that the combination of two pharmacological agents: the brain-permeable mTOR inhibitor RapaLink-1 and the brain-impermeable FKBP12 ligand RapaBlock could make brain-specific mTOR inhibition probably and may be beneficial to patients with BM of LUAD [50]. Future clinical trials are urgently awaited to verify the effects of mTOR inhibitors on the BM of LUAD because our findings suggest a potential association between RPTOR and the BM of LUAD.

Several limitations remain in the present study. First, the relationship between RPTOR expression and LUAD-BM, as well as their corresponding mechanism, was investigated in nude mice and zebrafish. These models, however, are somewhat defective. *RPTOR*-knockout transgenic mice may be a better alternative model to determine the role of RPTOR in LUAD-BM. Second, the in vitro BBB model only consisted of mixtures of hCMEC/D3 cells and astrocytes. Despite the construction ease and reliability, the growth of cells from transwell models is two-dimensional in nature, rendering impossible the real-life dynamic observation. Therefore, three-dimensional models of the human BBB are needed to better determine the mechanism of BM development. Finally, the mechanism underlying the interaction between RPTOR and YY1 needs to be further explored.

### Supplementary Information

**Additional file 1: Fig. S1.** Relationship between high RPTOR expression and poor prognosis in patients with NSCLC. **Fig. S2.** Effects of RPTOR on the migration, invasion, and proliferation of NSCLC cell lines in vitro. **Fig. S3.** The RPTOR-promoted NSCLC metastasis through the SPHK2 signaling pathway. **Fig. S4.** ABC294640 inhibition of the enhanced proliferation, migration and invasion of NSCLC cell lines induced by RPTOR. **Fig. S5.** The successfully constructed Zebrafish model with RPTOR overexpressed or knocked down.

## Data Availability

All data generated in this study are included in this article and its supplementary files.
