# Correction: RPTOR blockade suppresses brain metastases of NSCLC by interfering the ceramide metabolism via hijacking YY1 binding

**DOI:** 10.1186/s13046-024-02961-9

**Published:** 2024-02-02

**Authors:** Ying Lin, Yun Wu, Qiangzu Zhang, Xunwei Tu, Sufang Chen, Junfan Pan, Nengluan Xu, Ming Lin, Peiwei She, Gang Niu, Yusheng Chen, Hongru Li

**Affiliations:** 1 Department of Respiratory and Critical Care Medicine, Shengli Clinical Medical College, Fujian Medical University, Fujian Provincial Hospital, Fuzhou, 350001 Fujian China; 2 Department of General Practice Medicine, Fujian Provincial Hospital, Fuzhou, 350001 China; 3 The High Performance Computing Research Center, Institute of Computing Technology, Chinese Academy of Sciences, Beijing, 100095 China; 4 The Centre for Experimental Research in Clinical Medicine, Fujian Provincial Hospital, Fuzhou, 350001 Fujian China; 5 Fujian Provincial Key Laboratory of Medical Big Data Engineering, Fujian Provincial Hospital, Shengli Clinical College of Fujian Medical University, Fuzhou, 350001 Fujian China

**Correction: *J Exp Clin Cancer Res*** **43, 1 (2024)**

10.1186/s13046-023-02874-z

Following publication of the original article [1], the authors spotted an error in the author list. Ying Lin, Yun Wu and Qiangzu Zhang should be captured as equal contributors.

The correction does not affect the overall result or conclusion of the article. The original article has been corrected.
