# Supplementary material for: RPTOR blockade suppresses brain metastases of NSCLC by interfering the ceramide metabolism via hijacking YY1 binding
Source: J Exp Clin Cancer Res. 2024 Jan 2;43:1. doi: 10.1186/s13046-023-02874-z (PMC10759737; doi:10.1186/s13046-023-02874-z)
Supplement: Supplementary file 1 — Additional file 1: Fig. S1. Relationship between high RPTOR expression and poor prognosis in patients with NSCLC. Fig. S2. Effects of RPTOR on the migration, invasion, and proliferation of NSCLC cell lines in vitro. Fig. S3. The RPTOR-promoted NSCLC metastasis through the SPHK2 signaling pathway. Fig. S4. ABC294640 inhibition of the enhanced proliferation, migration and invasion of NSCLC cell lines induced by RPTOR. Fig. S5. The successfully constructed Zebrafish model with RPTOR overexpressed or knocked down. [file 13046_2023_2874_MOESM1_ESM.docx]

**
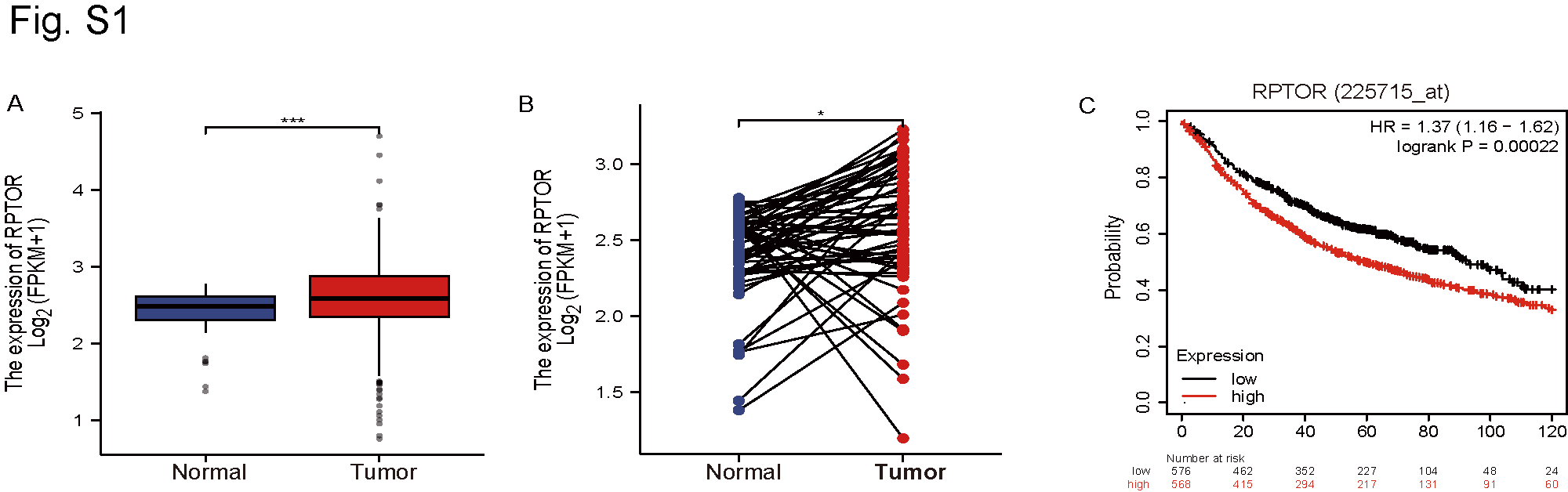
**

**Fig. S1 Relationship between high RPTOR expression and poor prognosis in patients with NSCLC.** (A, B) Results from the TCGA database showed that the level of RPTOR mRNA, as determined by analysis of transcriptome sequencing data, was significantly higher in 535 LUAD tissues than in 59 peritumoral tissues and more markedly elevated in 57 NSCLC tissues than in their paired peritumoral tissues. (C) Online Kaplan-Meier Plotter analysis of the above TCGA database showed that the survival of NSCLC patients with high RPTOR expression was significantly shorter than the survival of NSCLC patients with low RPTOR expression.


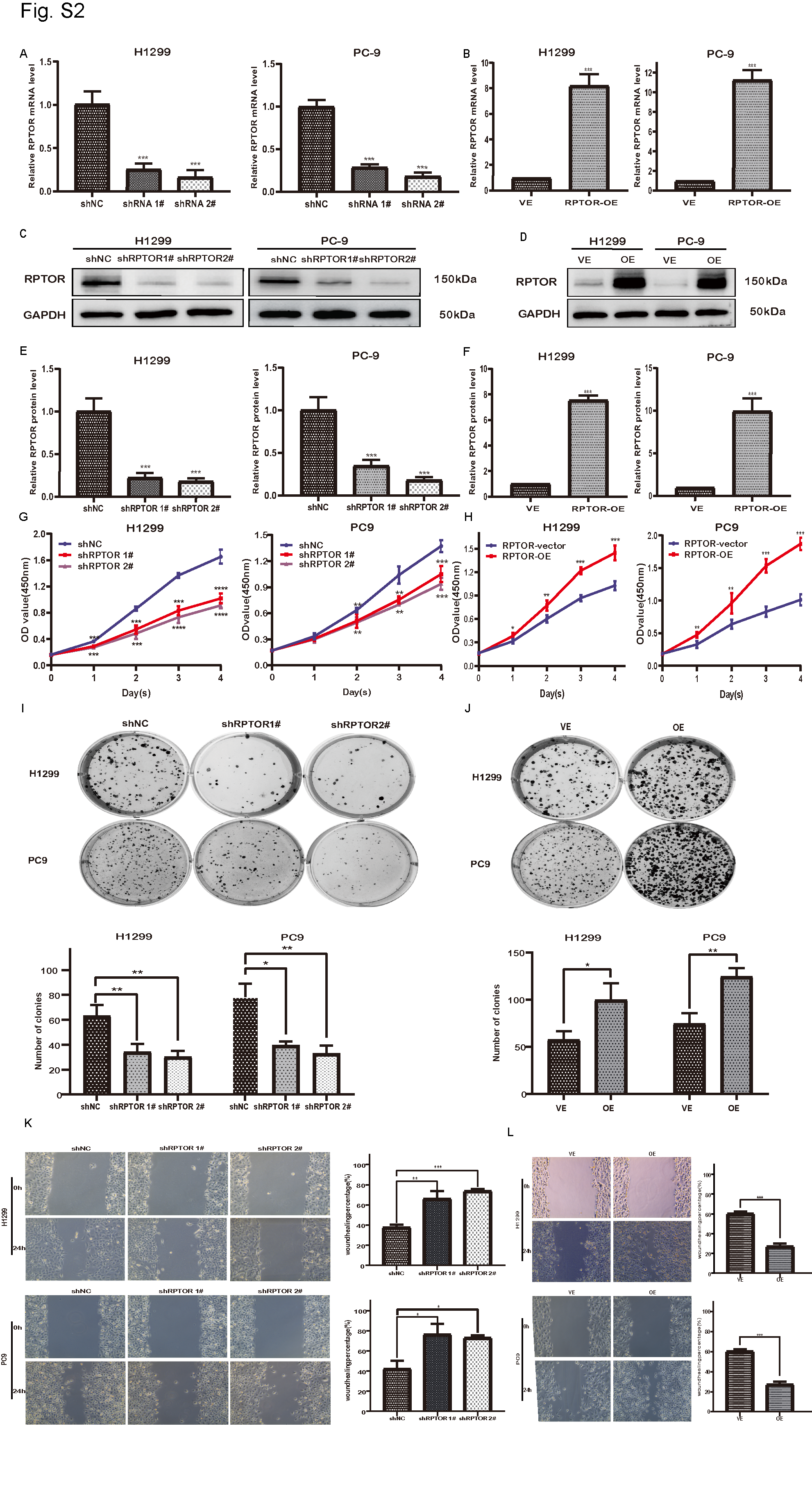


**Fig. S2 Effects of RPTOR on the migration, invasion, and proliferation of NSCLC cell lines in vitro**. (A-F) Levels of RPTOR mRNA and protein, as determined by qRT-PCR and western blotting, in lung cancer cell lines were stably transformed for RPTOR knockdown or overexpression. Compared with their respective negative controls, RPTOR mRNA, protein and relative quantitative assessment were significantly reduced by RPTOR knockdown, but significantly increased by RPTOR overexpression. (G-L) CCK8 and clone formation assays showed that the proliferation of lung cancer cells was significantly reduced by RPTOR knockdown, but significantly enhanced by RPTOR overexpression. Wound healing tests showed that would healing ability was reduced by RPTOR knockdown, but enhanced by RPTOR overexpression.


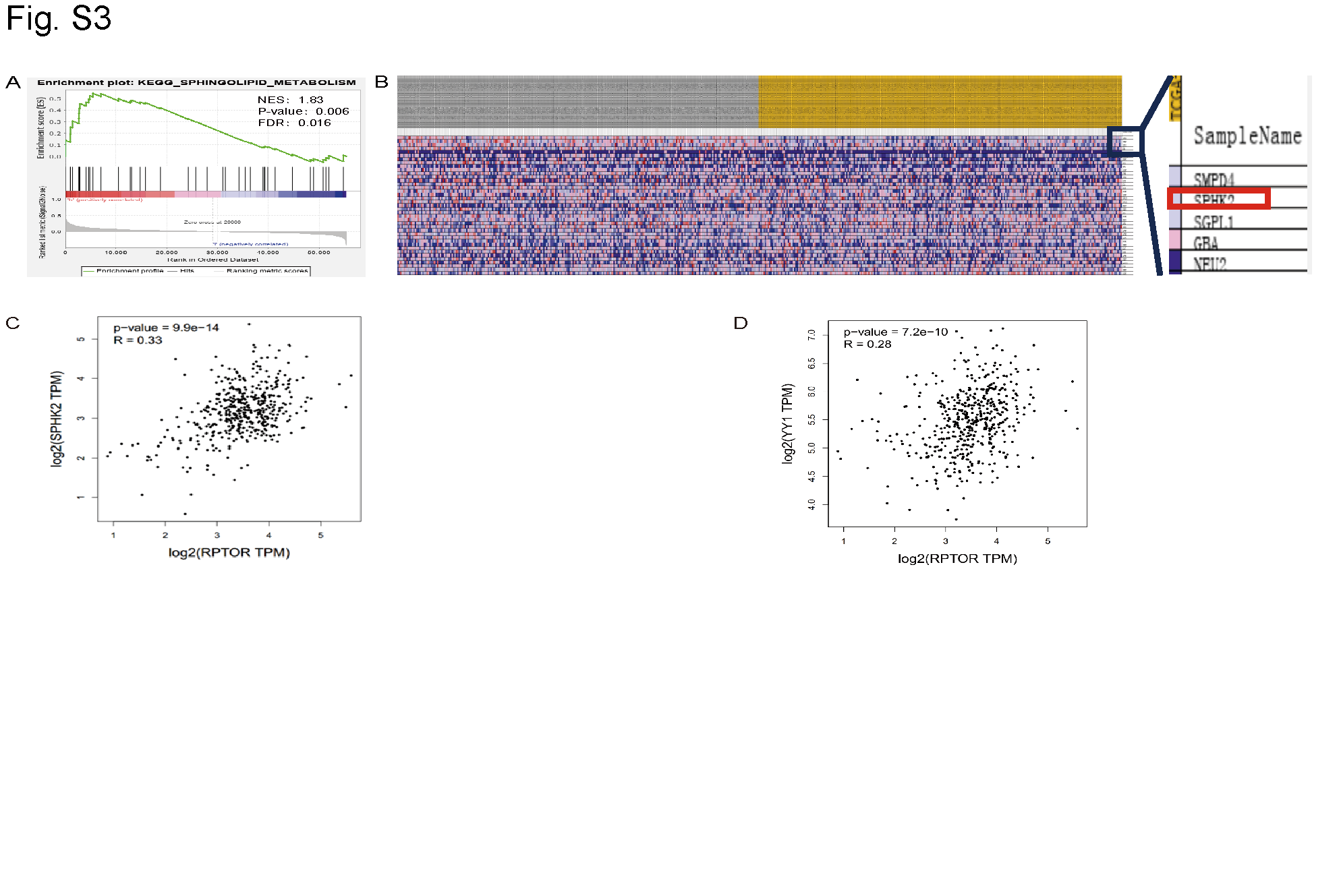


**Fig. S3 The RPTOR-promoted NSCLC metastasis through the SPHK2 signaling pathway.** (A, B) GSEA and KEGG pathway analysis of RNA sequencing results from LUAD patients with and without BM, showing that RPTOR was positively associated with the sphingomyelin pathway, with SPHK2 being the key factor in this pathway. Sphingomyelin consisted mainly of ceramide. (C, D) GEPIA database prediction of the correlation coefficients (R) between RPTOR and SPHK2 (0.33) and between RPTOR and YY1 (0.28).


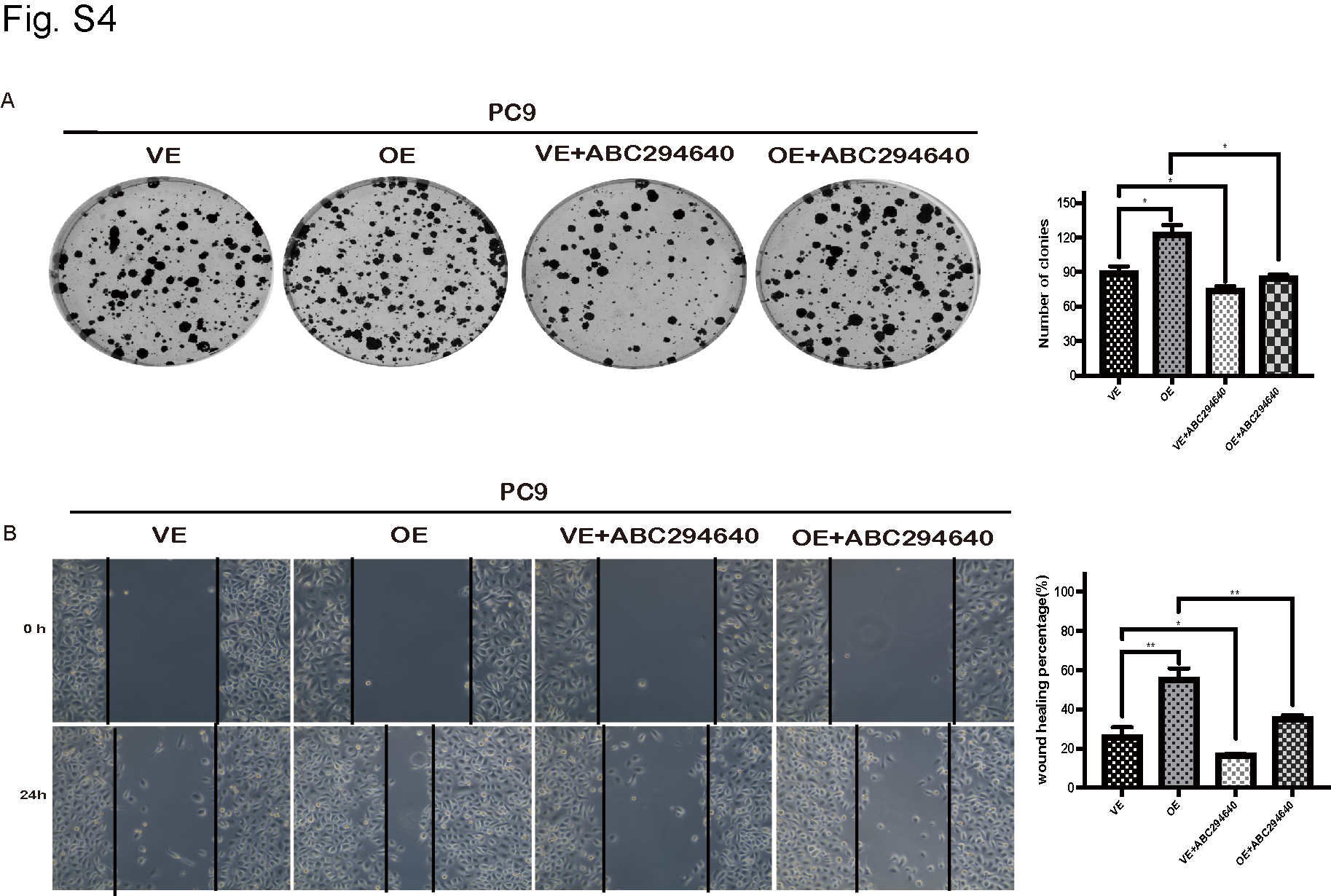


**Fig. S4 ABC294640 inhibition of the enhanced proliferation, migration and invasion of NSCLC cell lines induced by RPTOR.** (A, B) Clone formation and wound healing assays, showing that ABC294640 inhibited the proliferation of NSCLC cell lines with RPTOR overexpression, as well as reducing wound healing ability.


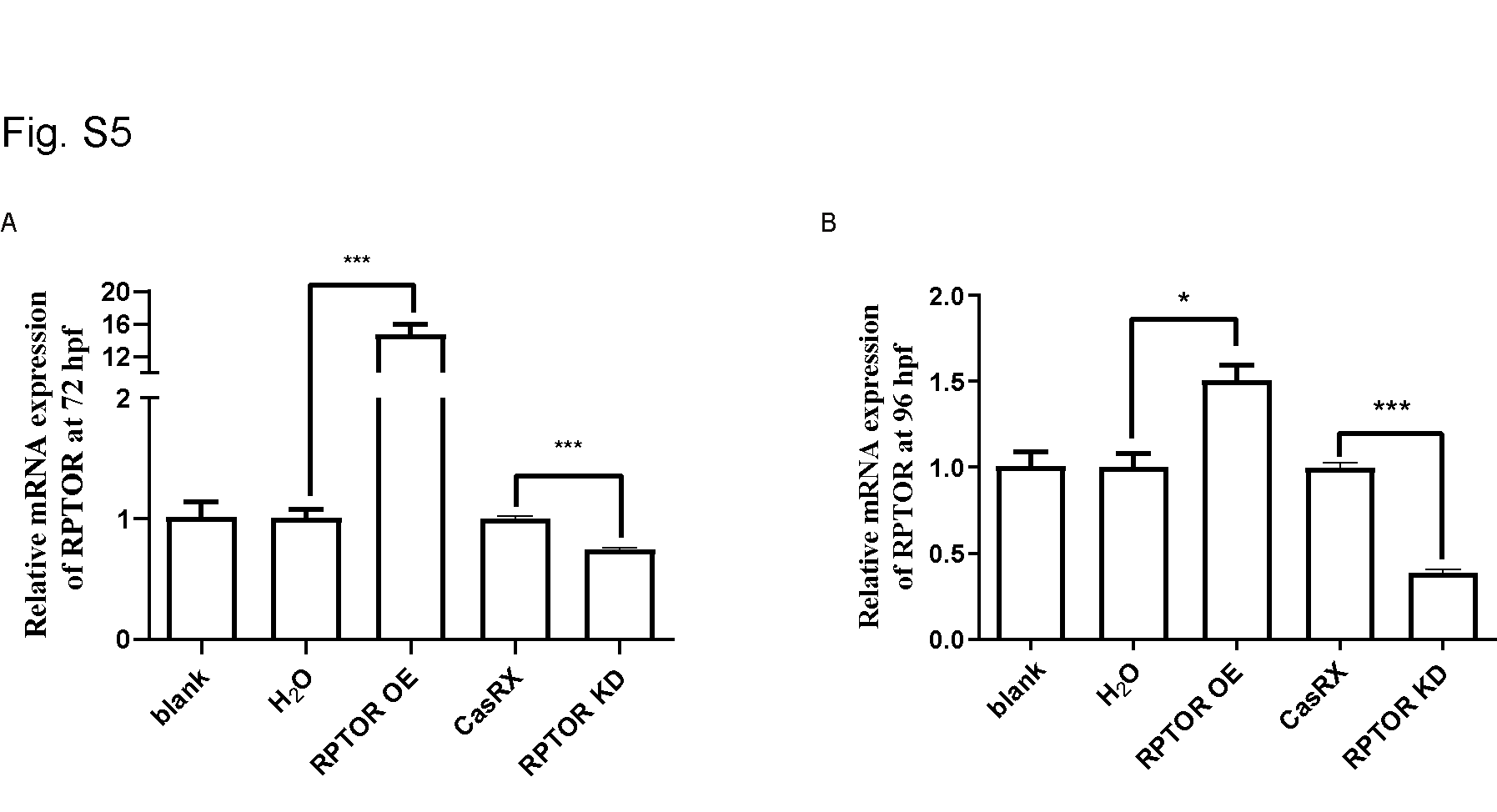


**Fig. S5 The successfully constructed Zebrafish model with RPTOR overexpressed or knocked down.** (A, B)We set the size of zebrafish eggs as 0 hpf (high power field). We detected relative mRNA expression of RPTOR from each group at 72 hpf and 96 hpf. According to the results of quantitative fluorometric assay, RPTOR-knockdowned group (RPTOR KD) had more significant difference in RPTOR expression than its CasRx-injected control group (CasRx) both at 72 hpf and at 96 hpf; compared with its H_2_O-injected control group (H_2_O), the difference of RPTOR-overexpressed group (RPTOR OE) was much more significant at 72 hpf and some at 96 hpf.
